# PLP2 of Mouse Hepatitis Virus A59 (MHV-A59) Targets TBK1 to Negatively Regulate Cellular Type I Interferon Signaling Pathway

**DOI:** 10.1371/journal.pone.0017192

**Published:** 2011-02-18

**Authors:** Gang Wang, Gang Chen, Dahai Zheng, Genhong Cheng, Hong Tang

**Affiliations:** 1 Key Laboratory of Infection and Immunity, Institute of Biophysics, Chinese Academy of Sciences, Beijing, China; 2 Department of Microbiology, Immunology and Molecular Genetics, University of California Los Angeles, Los Angeles, California, United States of America; 3 Research Network of Immunity and Health, Beijing Institutes of Life Science, Chinese Academy of Sciences, Beijing, China; 4 Graduate University, Chinese Academy of Sciences, Beijing, China; Yonsei University, Republic of Korea

## Abstract

**Background:**

Coronaviruses such as severe acute respiratory syndrome (SARS) coronavirus (SCoV) and mouse hepatitis virus A59 (MHV-A59) have evolved strategies to disable the innate immune system for productive replication and spread of infection. We have previously shown that papain-like protease domain 2 (PLP2), a catalytic domain of the nonstructural protein 3 (nsp3) of MHV-A59, encodes a deubiquitinase (DUB) and inactivates IFN regulatory factor 3 (IRF3) thereby the type I interferon (IFN) response.

**Principal Findings:**

Here we provide further evidence that PLP2 may also target TANK-binding kinase-1 (TBK1), the upstream kinase of IRF3 in the IFN signaling pathway. Overexpression experiments showed that PLP2 deubiquitinated TBK1 and reduced its kinase activity, hence inhibited IFN-β reporter activity. Albeit promiscuous in deubiquitinating cellular proteins, PLP2 inactivated TBK1 and IFN-β response in TNF receptor associated factor 3 (TRAF3) deficient cells, suggesting that targeting TBK1 would be sufficient for PLP2 to inhibit IRF3 activation. This notion was further supported by *in vitro* kinase assays, in which prior treatment of TBK1 with PLP2 inhibited its kinase activity to phosphorylate IRF3. Intriguing enough, results of PLP2 overexpression system and MHV-A59 infection system proved that PLP2 formed an inactive complex with TBK1 and IRF3 in the cytoplasm and the presence of PLP2 stabilized the hypo-phosphorylated IRF3-TBK1 complex in a dose-dependent manner.

**Conclusions:**

These results suggest that PLP2 not only inactivates TBK1, but also prevents IRF3 nuclear translocation hence inhibits IFN transcription activation. Identification of the conserved DUB activity of PLP2 in suppression of IFN signaling would provide a useful clue to the development of therapeutics against coronaviruses infection.

## Introduction

The innate immune system senses microbial infection and initiates counteractive response through evolutionary conserved pattern recognition receptors (PRRs) [1]–[3]. At least three classes of PRRs have been identified, designated Toll-like receptors (TLRs), retinoic acid-inducible gene I (RIG-I)-like helicases (RLHs) and nucleotide-oligomerization domain (NOD)-like receptors (NLRs). In response to virus infection, these receptors detect viral pathogen-associated molecular patterns (PAMPs) to elicit production of type I interferons (IFNs) and pro-inflammatory cytokines [4], [5]. These sensors, either on cell surface or in cytoplasm, usually require different adaptor molecules, such as TRIF, MyD88 or Cardif [6]–[9], for activation of two inhibitor of NF-κB kinase (IKK) homologues, namely TANK-binding kinase-1 (TBK1) and IKKε [10], [11]. Recent studies also indicate that a common TNF receptor associated factor 3 (TRAF3) adaptor complex is essential in the activation of TBK1 and IKKε for the production of IFNs [12], [13]. Activated TBK1 phosphorylates IFN regulatory factor 3 (IRF3), which then translocates to the nucleus and initiates transcription activation of IFN genes [14]. Secreted IFN further activates its down-stream signaling pathway, including phosphorylation of the tyrosine residues of the Janus kinase (JAK) and signal transducers and activators of transcription (STAT) proteins, to initiate anti-viral related genes expression [15].

Ubiquitination is to covalently conjugate the ubiquitin molecule(s) to the target proteins. There are seven lysine (K) residues within ubiquitin, and ubiquitination chains involving these different K play important roles in regulation of diverse fates of proteins. For example, K48-linked poly-ubiquitination usually leads to 26S proteasomal degradation of the modified proteins, whereas K63-linked ubiquitination often involves in signaling activation of numerous molecules. A large body of evidence has indicated that ubiquitination is critical for IFN induction. K63-linked ubiquitination of RIG-I by an E3 ubiquitin ligase TRIM25 is necessary and sufficient to trigger the downstream signaling cascade to produce IFN [16]. K63-linked autoubiquitination of TRAF3, an E3 ubiquitin ligase *per se*, is required in the activation of IFN signaling [13], [17]. TANK (TRAF family member-associated NF-κB activator), a scaffold protein of TBK1 and IKKε, is also reported to be poly-ubiquitinated through TRAF3- and Ubc13-dependent K63 linkage [18]. A recent study identifies that another E3 ligase, Nrdp1, can enhance the K63-linked ubiquitination and activation of TBK1 [19]. To keep the IFN activation in balance, there are a set of different cellular deubiquitinases, such as A20, CYLD, YopJ and deubiquitinating enzyme A (DUBA), that maintain the activation homeostasis of each essential checkpoint factors aforementioned [17], [20]–[23]. However, making the situation more complicated, ubiquitination does not always provide activation signal for IFN induction. For example, RBCK1, TRIM21 and a Cullin-based ubiquitin ligase are identified to induce poly-ubiquitination of IRF3, which leads to proteasomal degradation and inactivation of IRF3 [24]–[26].

Numerous viruses can precisely target the innate immune signaling pathway for productive replication and spreading. Clinical evidence has revealed that SARS coronavirus (SCoV), a highly pathologic Class II coronavirus, induces very low levels of IFN, indicating an evasion mechanism intrinsic to this family of viruses from the innate immune surveillance [27]–[29]. One possible mechanism is that the papain-like protease (PLpro) domain of the nonstructural protein 3 (nsp3) of SCoV can serve as a potent IFN antagonist by inhibiting the phosphorylation and nuclear translocation of IRF3 [30]. Our previous study further demonstrates that PLP2 domain of nsp3 of mouse hepatitis virus A59 (MHV-A59) encodes a deubiquitinase (DUB) domain conserved for the Class II coronaviruses, that can effectively deubiquinate IRF3 and prevent it from phosphorylation and nuclear translocation [31]. In this study, we further demonstrate that, in addition to IRF3, TBK1 is also targeted by PLP2 of MHV-A59. PLP2 not only deubiquitinates TBK1 and inactivates its kinase activity to phosphorylate IRF3, but also delays the dissociation of IRF3 from TBK1, thereby effectively attenuates IFN induction.

## Results

### The PLP2 domain of MHV-A59 nsp3 deubiquitinates TBK1

**Figure 1:**
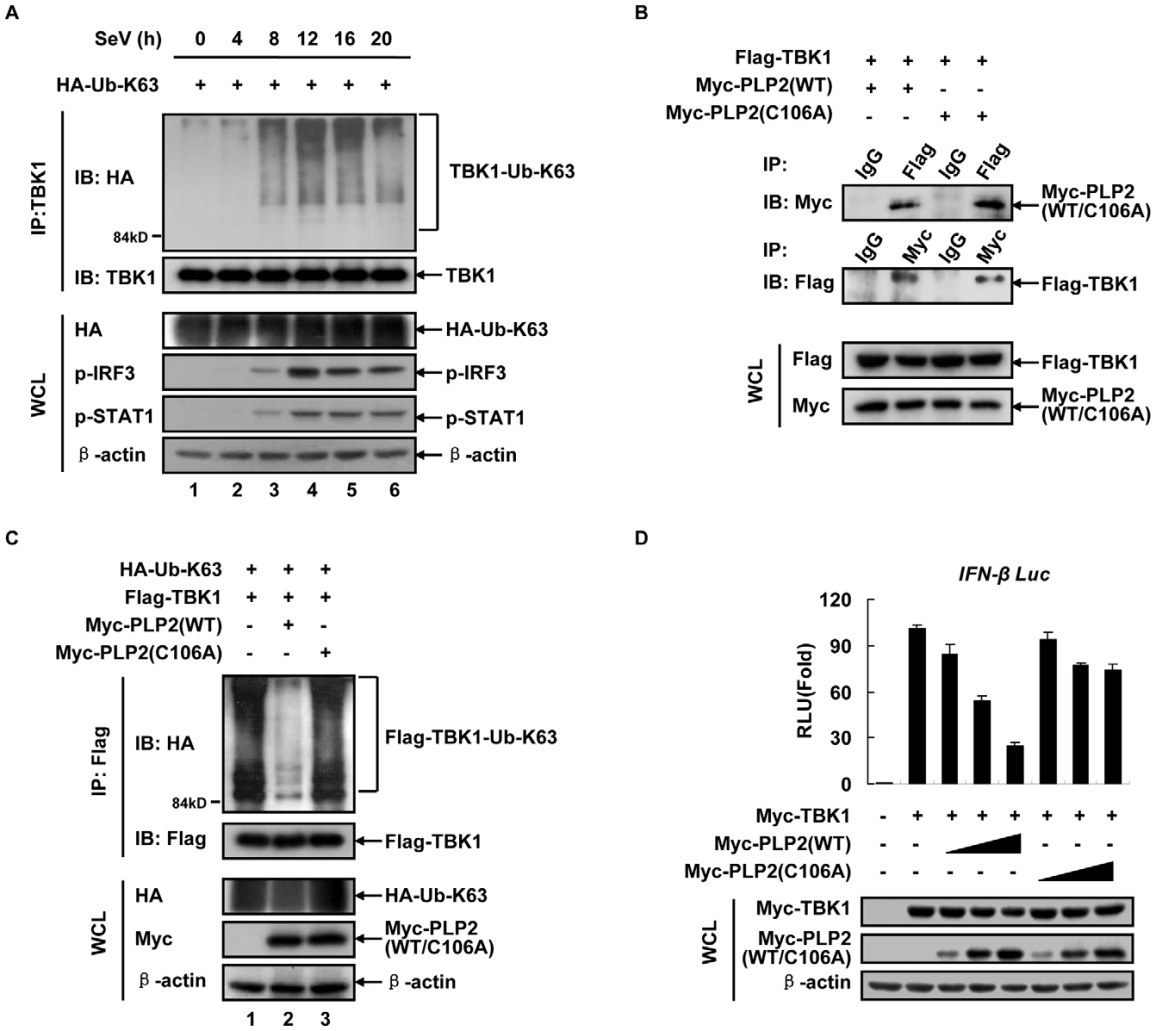
K63-linked ubiquitination involved in the process of TBK1 activation could be inhibited by MHV-A59 PLP2. (**A**) SeV infection induces K63-linked polyubiquitination of TBK1. HEK293T cells in 60 mm plates were transiently transfected with 3.6 µg HA-tagged ubiquitin K63 (HA-Ub-K63) expressing plasmids. At 24 h post transfection, cells were infected with SeV (HA titer 1∶25). At indicated time post infection, ubiquitination status of the endogenous TBK1 was immunoblotted with anti-HA antibody after immunoprecipitated by anti-TBK1 antibody (3 µg, IP: TBK1). TBK1 was not apparently degraded after viral infection as similar amounts of TBK1 were immunoabsorbed on beads (IB: TBK1). The whole cell lysates (WCL) was immunoblotted with anti-HA antibody for ubiquitin expression and massive cellular ubiquitination (HA), and anti-β-actin antibody for input. Immunobloting with phosphor-IRF3 and phosphor-STAT1 specific antibodies showed activation of TBK1 after viral infection for time indicated (p-IRF3, p-STAT1). (**B**) PLP2 associates with TBK1. HEK293T cells transiently expressing Flag-tagged TBK1 (Flag-TBK1) and Myc-tagged PLP2 (Myc-PLP2, WT or C106A) were lysed and immuoprecipitated with anti-Flag or -Myc antibodies. The immunoprecipitates were SDS-PAGE resolved and immunoblotted with antibody indicated. Mouse IgG was used as IP controls for Myc or Flag antibodies. (**C**) PLP2 deconjugates K63-linked polyubiquitin chains on TBK1. HEK293T cells (in 35 mm plates) transiently transfected with plasmids (800 ng each) encoding Flag-TBK1, HA-Ub-K63 or Myc-PLP2 (WT or C106A) for 24 h. Whole cell lysates were immunoprecipitated with anti-Flag antibody (1 µg) and SDS-PAGE resolved precipitates were immunoblotted with anti-HA or -Flag antibodies, respectively (IP: Flag). The expression of the epitope-tagged exogenous proteins was verified with the indicated antibodies (WCL). (**D**) PLP2 inhibits TBK1-driven IFN-β promoter activities. *IFN-β-Luc* promoter reporter (50 ng) and pCMV-Renilla internal control (15 ng) plasmids were co-transfected with Myc-TBK1 (100 ng) and Myc-PLP2 (WT or C106A, in three doses of 50, 100 and 200 ng) into HEK293T cells (24 well plates). Dual luciferase activities were measured and normalized to Renilla luciferase activities 24 h post transfection. Fold activation over the sham vector (pCMV-Myc) was averaged from three independent experiments (mean±SD). Expression of the exogenous epitope-tagged proteins was verified with the indicated antibodies (WCL). Data are representative of at least three independent experiments.

We have previously reported that PLP2 of MHV-A59 nsp3 deubiquitinated and inactivated IRF3 to inhibit cellular IFN induction [31]. Because multiple regulatory molecules upstream of IRF3 in the IFN pathway are involved in ubiquitination and deubiquitination, especially due to the fact that TBK1 can be ubiquitinated by a cellular E3 ligase Nrdp1 [19], we were tempted to ask whether PLP2 could target TBK1 to suppress the antiviral IFN signaling. Ubiquitination of TBK1 seemed an efficient tactic to activate IFN response because the endogenous TBK1 was K63-linked poly-ubiquitinated at 8 h post Sendai virus (SeV) infection (Fig. 1A, top panel) and accompanied with phosphorylation of IRF3 and STAT1, the indications of the IFN production (Fig. 1A, panels 4–5). On the other hand, co-immunoprecipitation experiments showed that PLP2 and its enzyme-dead mutant PLP2-C106A [31] formed a complex with TBK1 (Fig. 1B). Further ubiquitination assay demonstrated that overexpressed TBK1 became K63-linked poly-ubiquitinated, which was effectively inhibited by a co-expressed PLP2 but not PLP2-C106A (Fig. 1C). This was also supported by result of ubiquitination assay in MHV-A59 infection system. Using SeV as a control, MHV-A59 infection resulted in no marked K63-linked ubiquitination of TBK1 in mouse embryonic fibroblast (MEF) cells (Fig. S1). Moreover, the luciferase reporter experiments showed that TBK1-driven IFN-β promoter activities were reduced by PLP2 in a dose-dependent manner, but not by PLP2-C106A (Fig. 1D). These results indicated that PLP2 retarded the activation of TBK1 through its DUB activity.

### Targeting TBK1 by PLP2 is sufficient to block IFN induction

**Figure 2:**
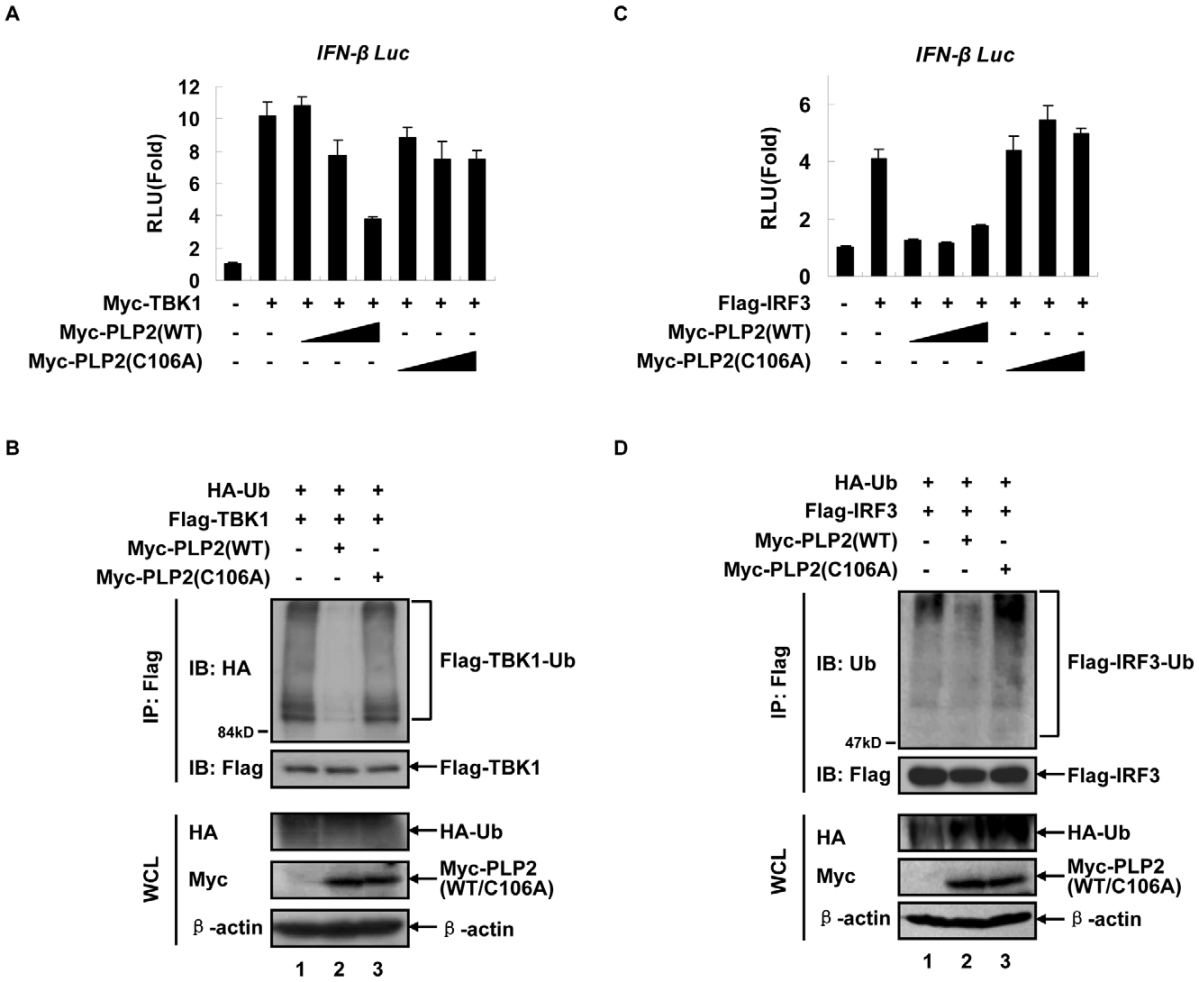
PLP2 inhibits IFN-β signaling by deubiquitinating both TBK1 and IRF3. (**A**) PLP2 inhibits TBK1-driven IFN-β promoter activities in *Traf3* ^−/−^ MEF cells. Luciferase assays were performed as in Fig. 1D except that *Traf3* ^−/−^ MEF cells (in 24-well plates) were transfected with different amount of each plasmids: 150 ng for *IFN-β-Luc* reporter, 50 ng for Renilla, 200 ng for Myc-TBK1 and increasing doses (100, 200 and 400 ng) for Myc-PLP2 (WT or C106A). Fold activation over the sham vector (pCMV-Myc) was averaged from three independent experiments (mean±SD). (**B**) PLP2 deubiquitinates TBK1 in *Traf3* ^−/−^ MEF cells. Experiments were performed as in Fig. 1C except that *Traf3* ^−/−^ MEF cells (in 10 cm plates) were transfected with 8 µg of each plasmid for Flag-TBK1, HA-Ub or Myc-PLP2 (WT or C106A) for 36 h before immunoprecipitation. (**C**) PLP2 inhibits IRF3-driven IFN-β promoter activities in *Tbk1* ^−/−^ cells. Experiments were carried out as in (A) except that plasmids expressing Flag-IRF3 and Myc-PLP2 (WT or C106A) were co-transfected into *Tbk1* ^−/−^ cells. (**D**) PLP2 deubiquitinates IRF3 in *Tbk1* ^−/−^ cells. Experiments were performed as in (B) except that *Tbk1* ^−/−^ MEF cells were transfected with Flag-IRF3, HA-Ub and Myc-PLP2 (WT or C106A). Data are representative of at least three independent experiments.

PLP2 is a potent deubiquitinase that has a broad spectrum of cellular substrates as shown in Fig. 1C as well as in our previous report [31]. To exclude the potential non-specific effect by PLP2 on IFN induction, especially on those regulatory molecules upstream of TBK1 in the IFN signaling pathway, we firstly tested whether PLP2 would still inhibit TBK1-driven IFN-β promoter activities in *Traf3* ^−/−^ MEF cells. Overexpression of TBK1 in *Traf3* ^−/−^ cells could still efficiently activate IFN-β promoter, suggesting that autonomously activated TBK1 could bypass the requirement of the upstream receptor-adaptors signaling. TBK1-driven IFN-β reporter activity, however, was effectively inhibited by the co-expressed PLP2 but not PLP2-C106A (Fig. 2A). Decreased IFN-β promoter activities correlated well to the reduced poly-ubiquitination level of TBK1 by PLP2 in *Traf3* ^−/−^ cells (Fig. 2B). These results therefore suggested that deubiquitination of TBK1 and/or IRF3 by PLP2 would be sufficient to reduce IFN-β promoter activities.

**Figure 3:**
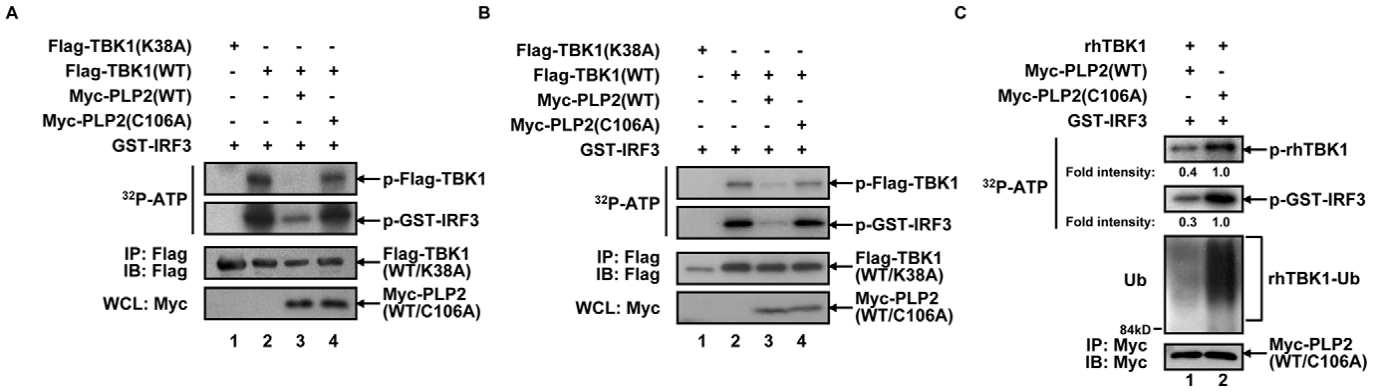
PLP2 inhibits IFN signaling by inactivating the kinase activity of TBK1. (**A**) PLP2 inhibits IRF3 phosphorylation by inactivating TBK1. Plasmids (800 ng) expressing Flag-TBK1 (WT or kinase dead mutant K38A) and Myc-PLP2 (WT or C106A) were co-expressed in HEK293T cells (in 35 mm plates). At 36 h post transfection, cells were lysed and Flag-TBK1 was immunoprecipitated with anti-Flag antibody. Immunoabsorbed Flag-TBK1 was incubation with recombinant GST-IRF3_131–426_ (1 µg) and γ-^32^P-ATP at 25°C for 30 min. Phosphorylation of proteins was resolved by SDS-PAGE and autoradiography. Expression of the exogenous proteins was verified with the indicated antibodies (WCL). (**B**) PLP2 inhibits TBK1 kinase activity in *Traf3* ^−/−^ MEF cells. The similar *in vitro* kinase assays were carried out as in (A) except that 8 µg of each plasmid expressing Flag-TBK1(WT or K38A) and Myc-PLP2 (WT or C106A) were co-transfected into *Traf3* ^−/−^ MEF cells (in 10 cm plates). (**C**) PLP2 inactivates the recombinant TBK1 by deubiquitination. An equal amount of recombinant TBK1 (500 ng) was incubated with Myc-PLP2 (WT or C106A) immunopurified from HEK293T cells at 37°C for 2 h. The kinase activities were measured as in (A). The deubiquitination efficiency of TBK1 was examined with anti-ubiquitin antibody and the amount of Myc-PLP2 (WT or C106A) used in each reaction was measured by anti-Myc antibody. Data are representative of at least three independent experiments.

Paradoxically, PLP2 can also reduce the ubiquitination level of IRF3 to diminish its ability in IFN induction [31]. Because IRF3 activation requires TBK1 [11], it is therefore desirable to delineate which factor, TBK1, IRF3 or both, is the primary target for PLP2. PLP2 action on IRF3 was apparently independent of TBK1, as PLP2 but not PLP2-C106A specifically inhibited IRF3-driven IFN-β promoter activities in *Tbk1* ^−/−^ cells (Fig. 2C). This was also correlated with the reduced poly-ubiquitination level of IRF3 by PLP2 in *Tbk1* ^−/−^ MEF cells (Fig. 2D). The apparent explanation for these results would be an advantageous strategy for coronavirus to evade the anti-viral line of defense through destructing multiple innate signaling components, e.g., TBK1 and IRF3. To determine which step of deubiquitination is causative, we first measured whether the kinase activities of TBK1 would be directly affected by PLP2 by *in vitro* kinase assays. Flag-TBK1 ectopically expressed in HEK293T cells in the presence of co-expressed PLP2 (WT or C106A) was affinity purified. The subsequent measurement of kinase activities using the purified C-terminal domain of IRF3 (GST-IRF3_131–426_) as substrate showed that the presence of PLP2 but not PLP2-C106A robustly inhibited the kinase activity of TBK1 in its autophosphorylation and IRF3 phosphorylation (Fig. 3A). The kinase activity of Flag-TBK1 immuno-purified from *Traf3* ^−/−^ MEF cells exhibited the similar dependence on PLP2 (Fig. 3B). Therefore, deubiquitinating TBK1 was sufficient for PLP2 to down-regulate IRF3 phosphorylation. Furthermore, we mixed the recombinant human TBK1 purified from insect cells with PLP2 (WT or C106A) immunopurified from HEK293T cells. Surprising enough, the recombinant TBK1 purified from insect cells was already ubiquitinated (Fig. 3C, the third panel), suggesting a conserved post-translational modification of TBK1. Pre-incubation of PLP2 reduced ubiquitination level of TBK1 and remarkably inhibited its kinase activity on IRF3 (Fig. 3C, the second panel). In contrast, addition of PLP2-C106A to the reaction did not interfere with TBK1 in phosphorylating IRF3. These results therefore suggested that deubiquitination of TBK1 by PLP2 would be sufficient to suppress type I IFN induction.

### PLP2 stabilizes the inactive TBK1-IRF3 complex in the cytoplasm

**Figure 4:**
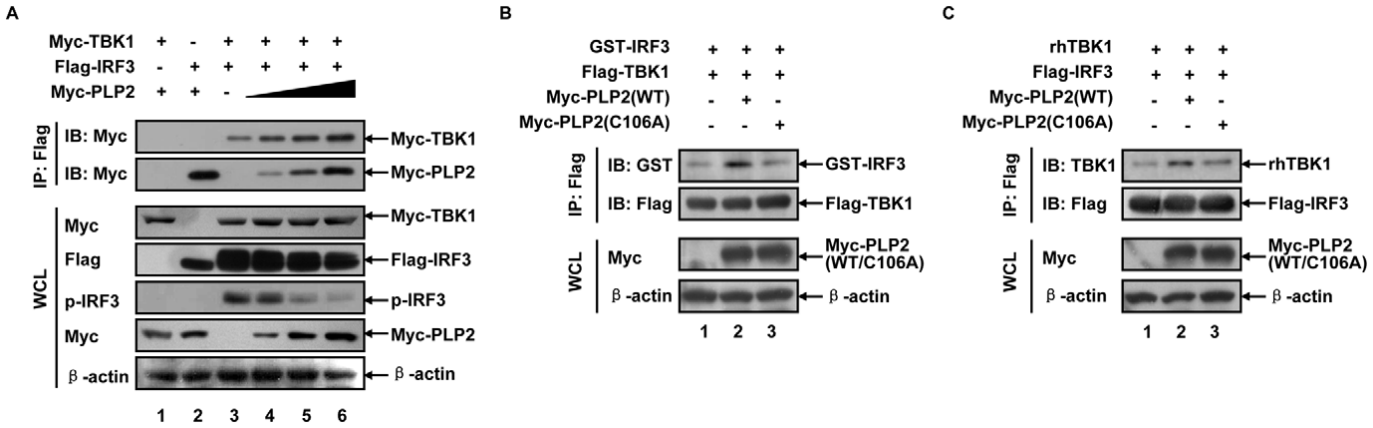
PLP2 stabilizes TBK1-IRF3 complex. (**A**) PLP2 inhibits the phosphorylation of IRF3 by inactivating TBK1 but stabilizes TBK1-IRF3 complex. A fixed amount of plasmids (800 ng each) for expressing Flag-IRF3 and Myc-TBK1, and an increasing amount (200, 400 and 800 ng) of Myc-PLP2 were co-transfected into HEK293T cells (in 35 mm plates). At 24 h post transfection, cells were lysed and immunoprecipitated with anti-Flag antibody. TBK1 and PLP2 associated with IRF3 were detected with anti-Myc antibody. Expression levels of the exogenous proteins were verified with the indicated antibodies. Anti-phosphor-IRF3 antibody was used to detect the activation status of IRF3 (WCL). (**B**) Hypo-ubiquitinated TBK1 bounds recombinant IRF3 more efficiently. Flag-TBK1 co-expressed with Myc-PLP2 (WT or C106A) was immuno-purified as in Fig. 3A and incubated with recombinant GST-IRF3_131–426_ (2 µg) in 1 mL lysis buffer at 4°C for 4 h. The formed TBK-IRF3 complex was then separated by centrifugation and SDS-PAGE resolved. The amount of IRF3 and TBK1 was immunoblotted with anti-GST and anti-Flag antibodies, respectively (IP: Flag). Expression of the exogenous proteins was verified with the indicated antibodies (WCL). (**C**) Hypo-ubiquitinated IRF3 interacts with TBK1 more efficiently. Flag-IRF3 in the presence of Myc-PLP2 (WT or C106A) was immunoprecipitated with anti-Flag antibody and each precipitate was incubated with recombinant human TBK1 as in (B). Pulled-down TBK1 by IRF3 was immunoblotted with anti-TBK1 antibody. Expression of the exogenous proteins was verified with the indicated antibodies (WCL). Data are representative of at least three independent experiments.

IRF3 is directly phosphorylated by TBK1 for transcription activation [11]. We have previously reported that IRF3 forms a complex with PLP2 [31]. In this study, we further demonstrated that PLP2 also formed a complex with TBK1. Therefore a tripartite complex containing TBK1/IRF3/PLP2 would exist in cells after infection of MHV-A59. To test this hypothesis, we co-expressed a fixed amount of Flag-IRF3 and Myc-TBK1 with elevated levels of Myc-PLP2 in HEK293T cells (Fig. 4A, WCL panel). Co-immunoprecipitation using Flag antibody showed that these three components formed an immuno-complex, and increasing amounts of Myc-PLP2 recruited more Myc-TBK1 into the complex (Fig. 4A, top two panels). Intriguing enough, anti-phospho-IRF3 antibody detected a decreasing phosphorylation of IRF3 (Fig. 4A, the fifth panel). It is therefore reasonable to speculate that the presence of PLP2 deubiquitinated and inactivated TBK1, which consequently decelerated the phosphorylation of IRF3 in the complex. Hypo-phosphorylated IRF3 would have a higher affinity to TBK1 as evidenced by more TBK1 associated with unphosphorylated IRF3. The presence of PLP2 likely stabilized the complex of TBK1 and IRF3 by inhibiting the dissociation of IRF3 from TBK1. To clarify if there would be any artifact in the abovementioned co-immunoprecipitation, we then mixed the enzyme and the substrate *in trans*. Immunopurified Flag-TBK1 co-expressing with Myc-PLP2 (WT or C106A) was incubated with purified GST-IRF3_131–426_. Immunoprecipitation with Flag antibody (Fig. 4B) demonstrated that GST-IRF3_131–426_ interacted with hypo-ubiquitinated TBK1 (coexpressed with wild type PLP2, lane 2) more efficiently than with hyper-ubiquitinated TBK1 (coexpressed with PLP2-C106A, lane 3). Similarly, if immunopurified Flag-IRF3 co-expressing with Myc-PLP2 (WT or C106A) was incubated with the recombinant TBK1 (Fig. 4C), TBK1 had a tendency to interact with hypo-ubiquitinated (coexpressed with wild type PLP2, lane 2) more efficiently than with hyper-ubiquitinated IRF3 (coexpressed with PLP2-C106A, lane 3).

**Figure 5:**
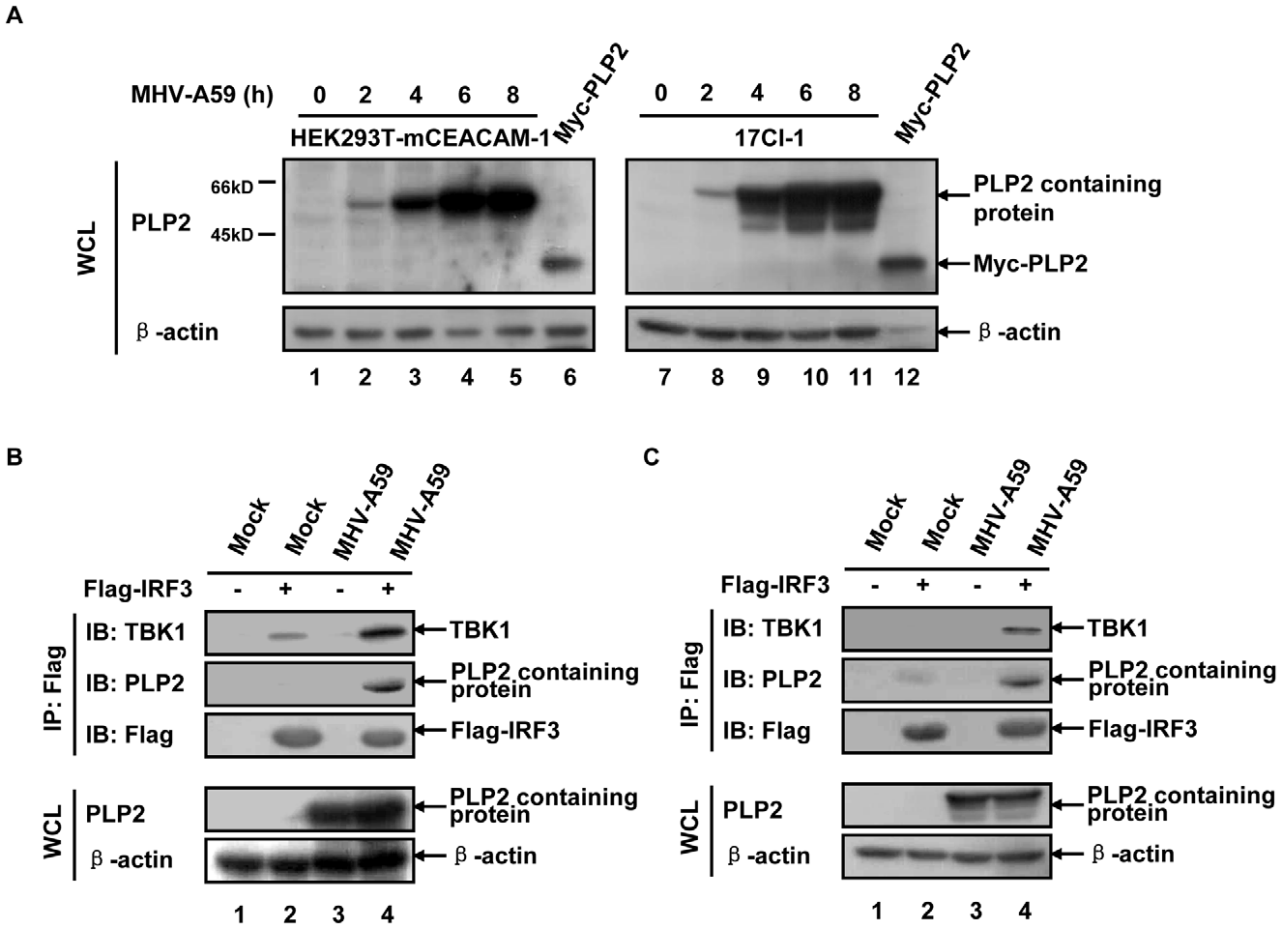
TBK1, IRF3 and PLP2 domain containing protein co-localize in one complex in MHV-A59 infected cells. (**A**) PLP2 domain containing protein is detected at 2 h post MHV-A59 infection. HEK293T-mCEACAM-1 cells or 17Cl-1 cells (in 35 mm plates) were infected with MHV-A59 (MOI = 5) for the time indicated, and cell lysates were immunoblotted with antiserum directed against MHV-A59 PLP2. Cell lysates of HEK293T ectopically expressing Myc-PLP2 was used as a positive control (lanes 6 and 12). (**B**) PLP2 containing protein stabilizes TBK1-IRF3 complex in MHV-A59 infected HEK293T-mCEACAM-1 cells. Plasmid encoding Flag-IRF3 or empty vector (1.2 µg) was transfected into HEK293T-mCEACAM-1 cells (in 35 mm plates). At 24 h post transfection, cells were simultaneously mock infected or infected with MHV-A59 for additional 10 h (MOI = 5) before lysed and immunoprecipitated with anti-Flag antibody. Endogenous TBK1 and PLP2 domain containing protein associated with Flag-IRF3 were immunoblotted with anti-TBK1 antibody or antiserum against PLP2 domain (IP: Flag). Expression levels of the exogenous proteins were verified with the indicated antibodies. (**C**) PLP2 containing protein stabilizes TBK1-IRF3 complex in MHV-A59 infected 17Cl-1 cells. Experiments were carried out as in (B) except with 17Cl-1 cells that expressing Flag-IRF3. Data are representative of at least three independent experiments.

The above results identified a trimeric complex of overexpressed PLP2 domain with TBK1 and IRF3. To investigate whether such a complex existed in MHV-A59 infected cells, we used a MHV-A59 permissive cell line 17Cl-1 and an engineered HEK293T cell line stably expressing MHV-A59 receptor (HEK293T-mCEACAM-1) to repeat the experiment mentioned above. Immunoblotting showed that antiserum directed against PLP2 domain could detect a 60 kD protein band as early as 2 h post MHV-A59 infection, indicating most likely the appearance of a cleavage product of nsp3 that contains PLP2 domain (Fig. 5A). To address if this PLP2 domain containing protein was also in the complex of TBK1-IRF3, we then overexpressed Flag-IRF3 in HEK293T-mCEACAM-1 cells or 17Cl-1 cells before MHV-A59 infection. Co-immunoprecipitation using Flag antibody yielded the results of Flag-IRF3 associating with the endogenous TBK1 and a viral protein positive for PLP2 antiserum in HEK293T-mCEACAM1 cells (Fig. 5B) and 17C1-l cells (Fig. 5C). Intriguing enough, although overexpressed IRF3 could recruit TBK1 (Fig. 5B and C, top panel lane 2), infection with MHV-A59 led to enhanced association of endogenous TBK1 with the complex (Fig. 5B and C, top panel lane 4). These results therefore strongly suggested that PLP2 domain that associated with TBK1/IRF3 complex may contribute to the suppressed IFN response via inactivation of TBK1/IRF3 by its DUB activity in MHV-A59 infected cells.

Therefore, by tempering the ubiquitination of both TBK1 and IRF3, and subsequent phosphorylation of both, PLP2 would provide a favorable steric conformation for inter-molecular interaction between inactivated TBK1 and IRF3. Such a stabilized complex would hinder the nuclear translocation of IRF3, thereby inhibit IFN induction. These results therefore provided a tentative model to explain why MHV-A59, in a broader sense for SCoV as well, fails to provoke robust IFN response either *in vivo* or *in vitro*.

## Discussion

The innate immune system is programmed to produce the type I IFN to deter viral infection, and ubiquitination is critically involved in the signaling process [25], [32]. On the other hand, during host-pathogen co-evolution, viruses have adopted effective gene programs to evade or subvert the innate immune system of the host cells [7], [25]. For example, the class II coronaviruses may adopt an unknown mechanism to shield MHV RNA from detection by intracellular sensor molecules [33], [34]. Papain-like protease of SCoV serves as a deubiquitinating enzyme [35] to block IRF3-mediated type I IFN induction [30]. This is supported by our previous work [31] that PLP2 of MHV-A59, a conserved class II coronavirus family member, can also specifically deubiquitinate and inhibit IRF3 activation, accountable for viral inhibition of cellular type I IFN production [36]. Other laboratory has also reported that MHV-A59 infection may partially limit the ability of IRF3 to function as a transcription factor [37]. Herein, we provide additional evidence on how class II coronaviruses subvert the innate defense. We have found that PLP2 of MHV-A59 can also target TBK1 to negatively regulate cellular type I IFN signaling pathway. It is the first evidence, to our best knowledge, that TBK1 activation is inhibited by a viral deubiquitinating enzyme.

TBK1 phosphorylates the C-terminal regulatory domain of IRF3 to activate IFN transcription [10], [11], [14]. Both factors are ubiquitinated to activate IFN signaling, with Nrdp1 on TBK1 [19], and an unknown E3 ligase on IRF3 [31], [36]. Our studies suggest that deubiquitination of TBK1 and/or IRF3 [31] by the viral PLP2 effectively reduce phosphorylation of TBK1 and IRF3, therefore IFN activation. Simultaneously or sequentially targeting TBK1 and IRF3 would be beneficial to MHV-A59 in spreading infection through effectively suppressing the type I IFN response. PLP2 is able to reduce TBK1 ubiquitination as well as its activity of phosphorylating IRF3 in *Traf3* ^−/−^ cells. We therefore tend to propose that TBK1 is the primary target for PLP2, and deubiquitination of TBK1 would be sufficient for PLP2 to inhibit IFN signaling. The conclusion that DUB activity of PLP2 of coronaviruses antagonizes IFN response is contradicted by a recent study in which DUB activity of PLP2 of another coronavirus HCoV-NL63 is not required to inhibit IFN response [38]. The most pronounced evidence came from the enzymatic mutants of PLP2 (C1678A and H1836A), which still possess dose-dependent inhibition of IFN-β promoter activity. This result indicates a possible catalytic activity-independent mechanism that acts to inhibit IFN induction by NL63 PLP2. However, in their study, inhibition by catalytic mutants of PLP2 is reduced compared to equivalent amounts of wild type PLP2. So, the possibility still could not be ruled out that enzymatic activity of PLP2 may also contribute to IFN antagonism. Furthermore, in their study, limited results proved that these two enzymatic mutants of PLP2 lost DUB activity completely. The ubiquitination level in cells expressed PLP2 C1678A seems lower than that in cells without PLP2. Another explanation would be the apparent difference of virus strains and host cells that elicit IFN responses. Restrained by the fact that TBK1 requires IRF3 to transmit signals, we were unable to delineate whether a reduced IFN response by PLP2 was due to deubiquitination of TBK1, IRF3, or both. Identification of the ubiquitin modification site(s) within TBK1 would help decipher the underlying mechanism.

The finding that PLP2 or PLP2 domain-containing protein stabilizes the TBK1-IRF3 complex is very intriguing. Most, if not all, evidences indicate that viruses inhibit type I IFN response through interfering with the inter-molecular interaction between IRF3 and TBK1/IKKε, such as VP35 of Ebola virus [39], V proteins of Paramyxovirus [40] and γ34.5 protein of Herpes simplex virus 1 [41]. This unexpected finding can be explained by the fact that PLP2 is a deubiquitinase *per se*. Removing polyubiquitin chains, possibly subsequent phosphate groups, would provide a favorable steric interface between TBK1 and IRF3 that enhances the inter-molecular interaction. Moreover, a deubiquinated TBK1 by PLP2 loses its kinase activity, thereby leads to more hypo- or un-phosphorylated IRF3 accumulated. Therefore, the presence of PLP2 would favor the formation of more cytosolic TBK1-IRF3 complex. In addition to shutting down the TBK1-IRF3 activation signaling, PLP2 can also help sequester IRF3 from nuclear translocation by stabilizing the TBK1-IRF3 complex in the cytosol, as we have observed previously [31]. The proposed inter-molecular interactions among PLP2, TBK1 and IRF3 in attenuating IFN signaling has not been able to be identified in another study [42]. This discrepancy would stem from the different PLP2 sequences used by two studies, with over 30 amino acids in the C-terminal region of PLP2 being truncated in the latter work.

In conclusion, MHV-A59 may take advantage of its PLP2 to negatively regulate the type I IFN signaling via targeting TBK1, the hub kinase in IFN signaling. As many different coronavirus proteins have been discovered to target various molecules of innate immune system [29], DUB activity of PLP2 may be one of the most effective arsenal for coronavirus to retard and/or escape from the type I IFN response. Understanding the underlying spatial and temporal regulation of innate immune response to MHV-A59 may shed a light on diagnostic and therapeutic discovery against coronavirus infection.

## Materials and Methods

### Cells and plasmids

TBK1 and TRAF3 knockout mouse embryonic fibroblast cells (*Tbk1* ^−/−^ and *Traf3* ^−/−^ MEF cells), wild-type MEF cells [43], [44], and HEK293T cells (ATCC, USA) were routinely maintained in DMEM (Hyclone, UT) supplemented with 10% FBS (PAA, Pasching, Austria) and 1% penicillin and streptomycin (Hyclone, UT). Cell line 17Cl-1 is a kind gift from Dr K Holmes (University of Colorado Health Sciences Center, USA). The overexpression constructs, pCMV-Myc-PLP2, its DUB mutant derivative, pCMV-Myc-PLP2-C106A, and pFlag-CMV2-IRF3 were described previously [31]. To construct bacterial expression vector pGEX-6P-2-PLP2, the DNA fragment encoding PLP2 was subcloned in frame into the *EcoRI* and *XhoI* sites of pGEX-6P-2 vector (GE Healthcare). Full-length human *Tbk1* gene was PCR subcloned into different pCMV vectors (Clontech) in between *SalI* and *NotI* sites, to generate plasmids of Flag- or Myc-tagged TBK1. Wild-type ubiquitin expression vector pRK5-HA-Ub and arginine substitutions of all lysine residues except for position 63 (designated as Ub-K63) [45], were provided by Dr K Lim (National Neuroscience Institute, Singapore). Bacterial expression vector, pGEX-4T-1-IRF3-131C [46], that expressing a GST-tagged C-terminal domain of IRF3 (GST-IRF3_131–426_), was kindly provided by Dr I. Rogatsky (Weill Medical College of Cornell University, USA). Full-length mouse *Ceacam-1* gene was created by PCR and was subcloned in frame into pBABE-puro vector in between *EcoRI* and *SalI* sites, to generate MHV receptor (MHVR) expression vector pBABE-puro-mCEACAM-1. To establish cell lines stably expressing MHVR, HEK293T cells seeded in a 12-well plate were transfected with 1.6 µg pBABE-puro-mCEACAM-1, and the stable clone was selected by 20-fold dilution in the presence of 1 µg/mL puromycin.

### Antibodies, reagents and virus stock

Antibodies specific to IRF3, ubiquitin, HA and GST were purchased from Santa Cruz Biotech (Santa Cruz, CA), Flag (M2) and β-actin from Sigma-Aldrich (St Louis, MO), Myc from Shanghai Genomics (Shanghai, China), phosphorylated STAT1, phosphorylated IRF3 and TBK1 were from Cell Signaling Tech (Beverly, MA). HRP conjugated goat anti-mouse IgG light chain specific secondary antibody was from Jackson Immunoresearch (West Grove, PA). Recombinant human TBK1 purified from baculovirus expression system was purchased from Invitrogen (Carlsbad, CA). γ-^32^P- ATP was obtained from FuruiBio (Beijing, China). All other fine chemicals were from Sigma-Aldrich (St Louis, MO). Expansion of Sendai virus (SeV) and MHV-A59 and infection experiments were described previously [31]. The mouse antiserum against MHV-A59 PLP2 domain was prepared by CoWin Biotech (Beijing, China) using the full length recombinant PLP2 as the immunogen.

### Transfection, co-immunoprecipitation and luciferase assays

Transient transfection of HEK293T cells with indicated plasmids was performed routinely with calcium phosphate method. Lipofectamine 2000 (Invitrogen) was used to transfect plasmids into other cells. For co-immunoprecipitation, cells were lysed with lysis buffer (25 mM HEPES, pH 7.5, 150 mM NaCl, 1% TritonX-100, 1 mM EDTA, 1 mM PMSF, 10 mM NaF, 1 mM Na_3_VO_4_ and 1× protease inhibitor cocktail) on ice at 24 h post transfection. Protein complexes were immunoprecipitated with indicated antibodies (IP) and separated by SDS-PAGE for immunoblotting analysis with antibodies (IB) as indicated. To detect ubiquitination of desired proteins, cells were collected and first sonicated in the denaturing buffer (lysis buffer plus 1% SDS and 1% β-mercaptoethanol) followed by boiling for 5 min. The lysates were then diluted with 10-fold lysis buffer before immunoprecipitation. *IFN-β* luciferase reporter assays and statistic analyses were performed exactly as previously described [31].

### RT-PCR

Total RNA was extracted according to manufacturer's instruction with Trizol (Invitrogen). RT-PCR was performed using RT-PCR kit (Promega) according to manufacturer's manual with the following primers: mouse *β-actin* forward, 5′-GTCCCTCACCCTCCCAAAAG-3′; mouse *β-actin* reverse, 5′-GCTGCCTCAACACCTCAACCC-3′; SeV *N* gene forward, 5′- GATCGTTGGGAACTACATCCGAG-3′; SeV *N* gene reverse, 5′- GACAGGTAGGTGTCTATGAGGC-3′; MHV *SM* gene forward (CK4), 5′-TCGAGAAGTTAAATGTTA-3′; MHV *SM* gene reverse (PM147), 5′-AGAAAATCCAAGATACAC-3′ [47].

### *In vitro* kinase assay

For TBK1 kinase assays, HEK293T (in 35 mm plates) or MEF cells (in 10 cm plates) were transfected with plasmids (HEK293T cells 0.8 µg, MEF cells 8 µg) expressing Flag-tagged TBK1 or its kinase dead mutant TBK1-K38A, in the presence of Myc-tagged PLP2 or its C106A mutant (HEK293T cells 0.8 µg, MEF cells 8 µg). TBK1 protein was immunoprecipitated from cell lysates 36 h after transfection with Flag antibody-absorbed beads. The yielded beads were then mixed with purified GST-IRF3_131–426_ protein (1 µg) in 25 µL kinase assay buffer (25 mM HEPES pH7.4, 1 mM DTT, 50 mM KCl, 2 mM MgCl_2_, 2 mM MnCl_2_, 10 mM NaF, 1 mM Na_3_VO_4_, 25 µM ATP, 0.2 µCi/µL γ-^32^P-ATP). The reaction took place at 25°C for 30 min before boiled in 1× SDS-PAGE loading dye for 5 min. Proteins were separated in 10% SDS-PAGE and phospho-proteins were analyzed by autoradiography.

### *In vitro* DUB assays

To produce PLP2 DUB, plasmids expression Myc-tagged PLP2 or its C106A mutant (1.6 µg) was first transiently expressed in HEK293T cells (in 35 mm plates) followed by immunoprecipitation to 20 µL Myc antibody-absorbed beads. Recombinant human TBK1 (500 ng) was then incubated with PLP2 immobilized beads in 25 µL DUB buffer (50 mM HEPES, pH 7.5, 50 mM KCl, 2 mM MgCl_2_, 1 mM DTT) at 37°C for 2 h. The effect of PLP2 on deubiquitination of TBK1 was assessed by IB with anti-Ub antibody. To detect the effect of PLP2 on the kinase activity of TBK1, the reaction buffer was adjust to 30 µL kinase assay buffer and purified GST-IRF3_131–426_ protein (1 µg) was added to the system at 25°C for additional 30 min.

## Supporting Information

Figure S1 **MHV-A59 infection does not induce K63-linked ubiquitination of TBK1.** MEF cells in 10 cm plates were transiently transfected with 24 µg HA-tagged ubiquitin K63 (HA-Ub-K63) expressing plasmids. At 24 h post transfection, cells were infected with MHV-A59 (MOI = 5) or SeV (HA titer 1∶25). At indicated time post infection, ubiquitination status of the endogenous TBK1 was immunoblotted with anti-HA antibody after immunoprecipitated by anti-TBK1 antibody (3 µg, IP: TBK1). The whole cell lysates (WCL) was immunoblotted with anti-HA antibody for ubiquitin expression and massive cellular ubiquitination (HA), and anti-β-actin antibody for input. Immunobloting with phosphor-STAT1 specific antibodies showed activation of TBK1 after viral infection for time indicated (p-STAT1). Total RNA of infected cells was extracted and subjected to RT-PCR with specific primers for MHV *SM* gene, SeV *N* gene, and mouse *β-actin* to check the viability of viruses.
(TIF)
