# Supplementary figures and images for: PLP2 of Mouse Hepatitis Virus A59 (MHV-A59) Targets TBK1 to Negatively Regulate Cellular Type I Interferon Signaling Pathway
Source: PLoS One. 2011 Feb 18;6(2):e17192. doi: 10.1371/journal.pone.0017192 (PMC3041802; doi:10.1371/journal.pone.0017192)

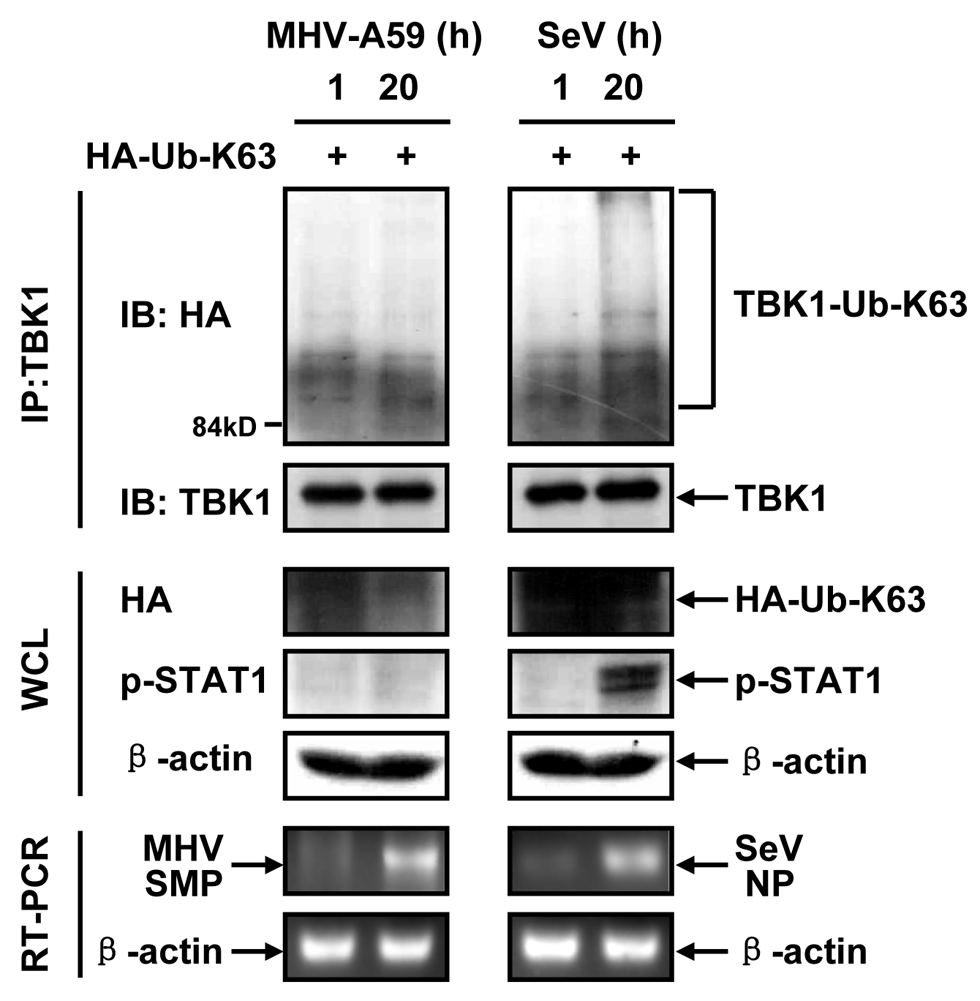

Supplement: Figure S1 — MHV-A59 infection does not induce K63-linked ubiquitination of TBK1. MEF cells in 10 cm plates were transiently transfected with 24 µg HA-tagged ubiquitin K63 (HA-Ub-K63) expressing plasmids. At 24 h post transfection, cells were infected with MHV-A59 (MOI = 5) or SeV (HA titer 1∶25). At indicated time post infection, ubiquitination status of the endogenous TBK1 was immunoblotted with anti-HA antibody after immunoprecipitated by anti-TBK1 antibody (3 µg, IP: TBK1). The whole cell lysates (WCL) was immunoblotted with anti-HA antibody for ubiquitin expression and massive cellular ubiquitination (HA), and anti-β-actin antibody for input. Immunobloting with phosphor-STAT1 specific antibodies showed activation of TBK1 after viral infection for time indicated (p-STAT1). Total RNA of infected cells was extracted and subjected to RT-PCR with specific primers for MHV SM gene, SeV N gene, and mouse β-actin to check the viability of viruses. (TIF) [file pone.0017192.s001.tif]
